# HSV Serologic Testing for Pregnant Women: Willingness to Be Tested and Factors Affecting Testing

**DOI:** 10.1155/2011/874820

**Published:** 2011-04-10

**Authors:** David A. Baker, Andrea Pressley, Lillian Meek, Reinaldo Figueroa, Barbara Yates, Lynn Dix

**Affiliations:** ^1^Division of Infectious Diseases, Department of Obstetrics, Gynecology, and Reproductive Medicine, School of Medicine, State University of New York at Stony Brook, Stony Brook, NY 11794-8091, USA; ^2^GlaxoSmithKline, Research Triangle Park, Durham, NC, USA

## Abstract

*Objective*. This prospective study was undertaken to evaluate pregnant women's willingness to undergo HSV type-specific serologic testing and factors affecting willingness in an obstetrics/gynecology ambulatory unit. *Methods*. At prenatal Visit 1, pregnant women (*n* = 303) with no history of HSV-2 were tested for HSV-1/HSV-2 before and after they received counseling on genital and neonatal herpes. *Results*. In both the Unwilling Subgroup and the group that changed from being willing to being unwilling, the most common reasons for choosing not to be tested were *not being at risk for genital herpes, being tested is too personal, and concern about what will be done with the results*. Of the 134 participants in the Willing/Tested Subgroup, 27 (20%) were HSV-2 seropositive and 81 (60%) were HSV-1 seropositive. *Conclusions*. These results support the feasibility of HSV serologic testing and counseling in pregnant women.

## 1. Introduction

An estimated 45 million people in the United States, including approximately 1 in 5 pregnant women (22%), have genital herpes caused by herpes simplex virus-2 (HSV-2) [1–3]. HSV-1 infection, also highly prevalent in the United States, affects approximately two thirds of pregnant women (63%) [3]. HSV infection in pregnancy is of particular concern because of one of its most devastating sequelae—neonatal herpes, which is associated with significant neurodevelopmental impairment or death in at least one third of infected babies [4]. Caused by intrapartum exposure of the fetus to HSV-2 or, less frequently, HSV-1, neonatal herpes has been estimated to affect approximately 1500 infants annually in the United States although recent findings suggest a higher incidence [5–7]. 

Means of reducing risk of HSV transmission to the neonate are directed at women with established, symptomatic infection: using antiviral prophylaxis during the last 4 weeks of pregnancy delivering via cesarean section instead of vaginally and limiting the use of invasive procedures in women shedding HSV during labor [4, 8]. Because these strategies do not address prevention of maternal HSV acquisition during pregnancy or subclinical HSV infections (~90% of HSV infections), they prevent only an estimated 20% to 30% of cases of neonatal herpes [9]. The majority of HSV infections in pregnancy are undiagnosed and are therefore not addressed by current preventative strategies [10]. For example, in a recent study of infants born to 252,474 mothers from January 1997 to June 2002 in a large US managed-care database, a prior diagnosis of HSV infection had been made in only 12% of mothers who gave birth to infants with neonatal HSV [6].

Because awareness of maternal HSV status is integral to efforts to prevent viral transmission to the neonate, improvement in diagnosis of genital herpes among pregnant women is warranted. Type-specific serological tests, which can detect HSV regardless of whether lesions are present, constitute the most effective means of diagnosing HSV. Many experts recommend routine HSV serologic testing during pregnancy [11–15] while others discourage it or do not practice routine testing on the basis of concerns such as the negative psychological impact of HSV testing and lack of cost-effectiveness [9, 16–20]. The current prospective study was undertaken to evaluate pregnant women's willingness to undergo HSV type-specific serologic testing, factors affecting their willingness, and the feasibility of HSV serologic testing and counseling in an obstetrics/gynecology ambulatory unit.

## 2. Materials and Methods

### 2.1. Sample

Pregnant women at least 18 years old and at 15 ± 3 weeks of gestation were eligible for the study if they did not have a known history of HSV-2 infection. All participants provided written informed consent.

### 2.2. Procedures

**Figure 1:**
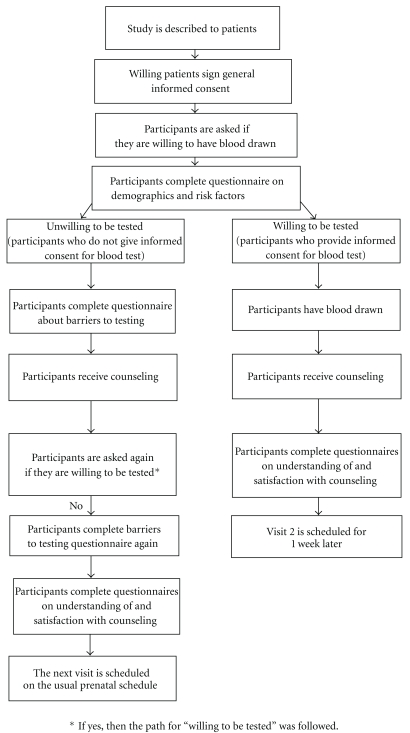
Study procedures for Visit 1.

An institutional review board approved the protocol for this prospective study conducted at a single study center in the Department of Obstetrics and Gynecology and Reproductive Medicine Health Science Center at the State University of New York at Stony Brook (Stony Brook, NY, USA). The study included two office visits, Visit 1 (the first prenatal visit; Figure 1) and Visit 2. At Visit 1, the study was described to patients. Patients were told that the study (1) involved their willingness to have a blood test to determine whether they were infected with the virus that causes genital herpes and the virus that causes cold sores and (2) would require a time commitment of approximately 1 hour at this visit and, possibly, an additional 20-minute office visit the next week. Those providing written informed consent to participate in the study were asked to complete a questionnaire on demographics and baseline clinical information. 

Blood was drawn for testing for HSV-1/HSV-2 from consenting participants via the FOCUS HerpeSelect 1 and 2 ELISA assay. Using a standardized script (the appendix), health care providers then counseled participants about genital herpes, its risks to the neonate, and the significance of positive and negative serologic tests for genital herpes. These participants, the Willing/Tested Subgroup, were asked to complete two brief, self-administered questionnaires addressing their understanding of the information presented during counseling and their satisfaction with counseling, respectively. Questionnaire items assessing participants' understanding were declarative statements about herpes with response options of *true *or *false*. Questionnaire items assessing participants' satisfaction with various aspects of counseling had response options of *very satisfied*, *satisfied*, *neutral*, *dissatisfied*, or *very dissatisfied*. 

Participants unwilling to be tested (the Unwilling Subgroup) were asked to complete a questionnaire addressing barriers to serologic testing. Questionnaire items, which corresponded to specific potential reasons for being unwilling to be tested, had multiple-choice response options: *strongly disagree*, *disagree*, *agree*, and *strongly agree*. Health care providers then counseled them about genital herpes with the same standardized script described above (the appendix). After they were counseled, participants were again asked if they were willing to be tested. If they agreed and provided informed consent, they were given the blood test. If they remained unwilling to be tested, they were asked to complete the questionnaire addressing barriers to serologic testing again and to complete the two self-administered questionnaires addressing participants' understanding of the information presented during counseling and their satisfaction with counseling, respectively. 

At Visit 2, which occurred approximately 1 week after Visit 1, participants who were serologically tested at Visit 1 were counseled about the results of their test and encouraged to discuss the results with their partners. Participants who were HSV-2 seropositive were also provided with an educational brochure on reducing risk of transmission of genital herpes and were asked if they were willing to take medicine to treat their herpes during pregnancy. Both seropositive and seronegative participants were given two self-administered questionnaires, adapted to participants' serostatus, that addressed their understanding of the information presented during counseling and their satisfaction with counseling, respectively.

### 2.3. Measures and Data Analysis

The primary endpoint was the percentage of participants who expressed willingness to be tested for HSV-1 and HSV-2 and who provided a blood sample. Other endpoints included the percentages of participants in the Unwilling Subgroup expressing particular reasons for being unwilling to be tested, HSV-1 and HSV-2 seroprevalence among those who provided a blood sample, responses to questions on the counseling questionnaires in the Willing/Tested Subgroup compared with the Unwilling Subgroup, the percentage of HSV-2 seropositive participants willing to accept antiherpes therapy during pregnancy, health care providers' ratings of the median time spent counseling participants, and the specialty of the health care provider who counseled participants. 

Some participants initially expressed a willingness to be tested for HSV but later became unwilling to be tested and declined to provide a blood sample. These participants were categorized as being unwilling to be tested and included in the Unwilling Subgroup. In *post hoc* summaries, reasons for not being tested and responses on the counseling questionnaires were summarized separately for the Unwilling Subgroup and the portion of this subgroup that changed from being willing to being unwilling.

All data were summarized with descriptive statistics. In addition, the Willing/Tested Subgroup was compared with the Unwilling Subgroup on demographic variables and sexual history with chi-square or Fisher's Exact tests and on responses to the counseling questionnaires with Fisher's Exact tests.

## 3. Results

### 3.1. Sample

The sample comprised 304 women who consented to participate in the study. Demographics and baseline clinical characteristics are described in Table 1. Participants' mean (SD) age was 29.3 (6.2) years. The majority (87%) of participants were white, 8% were black, and the remainder was of Asian heritage. Approximately two thirds (64%) of participants were employed part or full time, and most women (83%) reported having private or employer-provided health insurance. None of the participants reported a history of genital herpes infection, human immunodeficiency virus (HIV) infection, or a rash or viral illness since their last menstrual period. The majority of participants (82% to 92%, depending on the symptom) reported 0 episodes in the past 6 months of genital itching or redness, burning or tingling around genitals, thighs, or buttocks, pain with urination, discharge with urination, or vaginal discharge not associated with other symptoms. 

Three hundred and four (304) women enrolled in the study and were included in demographic summaries. However, the analyses on willingness to be tested and summaries of questionnaire responses were based on data from 303 women because 1 participant enrolled but prematurely withdrew before providing evaluable data on the questionnaires or willingness to be tested.

### 3.2. Willingness to Be Tested for HSV-2

At Visit 1, 134 of 303 participants (44%) were willing to be tested for HSV-1/HSV-2 and provided a blood sample (Willing/Tested Subgroup), and 169 (56%) were unwilling to be tested (Unwilling Subgroup). Although 249 of 303 participants (82%) initially indicated that they were willing to be tested, 115 (38% of the sample) changed from willing to unwilling and did not provide blood samples at Visit 1. Participants had to go to another location to get their blood drawn, and it was not part of their routine prenatal blood workup. These 115 participants were included in the Unwilling Subgroup. Of the participants initially unwilling to be tested, only 2 changed from being unwilling to being willing after counseling at Visit 1. The number of patients never willing to be tested was 52.

The Willing/Tested Subgroup did not differ from the Unwilling Subgroup with respect to most demographic variables or sexual history (Table 1). However, those willing to be tested were more likely than those unwilling to be tested to be married (65% versus 54%) and to have private/employer-provided health insurance (89% versus 79%) (*P* < .05 for frequency distributions of responses for marital status and insurance type) (Table 1).

In both the Unwilling Subgroup and the 52 patients never willing to be tested, the most common reason for choosing not to be tested was *not being at risk for genital herpes* (Table 2). In both the Unwilling Subgroup and the group of participants who changed from being willing to being unwilling, the most common reasons for choosing not to be tested were *not being at risk for genital herpes*, *being tested is too personal*, and *concern about what will be done with the results *(data not shown). Proportionally more participants who changed from willing to unwilling than participants in the Unwilling Subgroup as a whole cited these reasons for not wanting to be tested (% participants who strongly agreed/agreed: 85% versus 90% for *not at risk*, 53% versus 63% for *being tested is too personal*, and 51% versus 60% for *concern about what will be done with the results*) (differences not statistically tested). The frequency distribution of reasons for choosing not to be tested did not differ as a function of whether participants were asked about testing before or after they received counseling (data not shown).

### 3.3. HSV Seroprevalence

Of the 134 participants in the Willing/Tested Subgroup, 27 (20%) were HSV-2 seropositive and 81 (60%) were HSV-1 seropositive (Table 3). The percentages of participants testing both HSV-1 and HSV-2 positive, both HSV-1 and HSV-2 negative, HSV-1 positive and HSV-2 negative, and HSV-1 negative and HSV-2 positive were 11%, 31%, 49%, and 9%, respectively (Table 3).

### 3.4. Counseling

The median time commitment for counseling the blood draw on Visit 1 for participants who consented to the blood draw was 30 minutes per participant (range 20 to 35 minutes). Counseling was provided by a nurse for 70% of participants, by an obstetrician/gynecologist for 26%, and by another health care provider for 4%.

**Visit 1**

Participants' understanding of the information from counseling at Visit 1 was generally good (Table 4). The majority of participants answered each question correctly. Nevertheless, 15% of participants incorrectly answered that *you cannot spread herpes to your newborn at the time of delivery*. Results did not differ between the Willing/Tested Subgroup and the Unwilling Subgroup (Table 4) or between the latter subgroups and those who changed from willing to unwilling to be tested (data not shown).

Nearly all participants (≥99%) indicated that they were *satisfied* or *very satisfied* with each of the probed aspects of counseling. Results did not differ between the Willing/Tested Subgroup and the Unwilling Subgroup.

**Visit 2**

Participants' understanding of counseling information from Visit 2 was generally good regardless of serostatus (Table 4). The majority of participants answered each question correctly. Among participants who were HSV-2 seropositive, 100% agreed that it is important to use medicine to treat herpes during pregnancy, and all were willing to take a medicine during the last 4 weeks of pregnancy to treat herpes. Nearly all participants (≥99%) indicated that they were *satisfied* or *very satisfied* with each of the probed aspects of counseling.

## 4. Discussion

Previous studies involving patient surveys suggest that pregnant women perceive the value of prenatal screening for genital herpes and are willing to be tested [21, 22]. This study extends previous findings by being the first to determine whether participants who express a willingness to be tested also agree to provide a blood sample for serologic testing, to assess barriers to testing, and to assess demographic and clinical correlates of willingness and unwillingness to be tested. In this study conducted in a US obstetrics/gynecology ambulatory unit, 44% of 303 women who were given the opportunity to be tested for HSV-1/HSV-2 agreed to be tested and provided a blood sample at their first prenatal visit. These women agreed to be tested and provided a blood sample prior to receiving any counseling or other information from the practice about genital or neonatal herpes; they were simply asked whether they would be willing to have a blood test to determine if they were infected with the virus that causes genital herpes or the virus that causes cold sores.

Among the 56% of participants unwilling to be tested for HSV, the most common reasons for being unwilling to be tested were *not being at risk for genital herpes *(85%), *being tested is too personal *(53%), and *concern about what will be done with the results *(51%). Substantial proportions of this Unwilling Subgroup also cited other reasons for being unwilling including *test would take too much time *(34%), *do not want to know results *(31%), *fear of results *(27%), *having to tell my partner *(24%), and *no benefit to finding out results *(22%). The Unwilling Subgroup did not differ from those willing to be tested with respect to sexual history or demographic variables such as age, race, pretax income, or highest level of education. However, those willing to be tested were significantly more likely than those unwilling to be tested to be married and to have private/employer-provided health insurance. These reasons for and demographic correlates of being unwilling to be tested do not point to specific issues or factors that determine the majority of participants' willingness or unwillingness to be tested for HSV nor do they suggest a particular profile of the individual willing or unwilling to be tested. Rather, the data are consistent with the presence of heterogeneous, patient-specific determinants of willingness to be tested for HSV among pregnant women.

The proportion of the sample that actually provided a blood sample for determination of HSV serostatus (44%) was substantially lower than that initially indicating a willingness to be tested for HSV (82%). With those who initially were willing to be tested but changed to unwilling as with the Unwilling Subgroup as a whole, the most common reasons for being unwilling to be tested included *not being at risk for genital herpes *(90% of those providing reasons for being unwilling), *being tested is too personal *(63%), and *concern about what will be done with the results *(60%). As only 71 or 72 (depending on the reason) of 115 participants who changed from willing to unwilling provided reasons for being unwilling, these data may be unrepresentative and should be interpreted cautiously. Whether aspects of the study protocol affected willingness to provide a blood sample is not known. Participants had to go to another location to get their blood drawn, and it was not part of their routine prenatal blood workup. The latter factors may have influenced willingness to provide a blood sample. In addition, the questionnaire included several items on presence of baseline genitourinary signs and symptoms such as genital itching or redness, burning or tingling around genitals, thighs, or buttocks, pain with urination, discharge with urination, and vaginal discharge. Most participants reported absence of these symptoms. Possibly, the absence of these symptoms contributed to participants' perception of not being at risk from genital herpes and suggested to them that they would not benefit from serologic testing. In addition, participants had to go to another location to get their blood drawn, and it was not part of their routine prenatal blood workup. These factors may also have affected willingness to be tested.

Although participants generally showed good understanding and retention of the information imparted during counseling, being counseled about genital herpes and neonatal herpes appears not to have been effective in motivating participants who were initially unwilling to be tested to change their minds: only 2 of those initially unwilling to be tested decided to have their blood tested after the brief counseling session. The counseling did not affect the reasons for being unwilling to be tested: the frequency of citing reasons for being unwilling to be tested did not differ as a function of whether or not participants had received counseling. The counseling may have been ineffective because it did not address some of the most common reasons for not being willing to be tested: *being tested is too personal* and *concern about what will be done with the results*. The counseling information was specific to genital herpes and its potential effects on the neonate; it did not include information on confidentiality or other aspects of disposition of the test results. Perhaps inclusion of such information would be useful in reducing unwillingness to be tested, a possibility that warrants further study.

HSV-2 seroprevalence in this sample from a single obstetrics/gynecology ambulatory unit was 20%, a result consistent with the prevalence of HSV-2 in US women generally and with US population-based data showing HSV-2 seroprevalence of 22% among pregnant women [1–3]. Likewise, the HSV-1 seropositivity rate of 60% in this study is consistent with the US population rate of 63% [3]. None of the women who tested positive for HSV-2 were aware of their HSV-2 serostatus prior to enrollment in the study. (Awareness of prior HSV-1 serostatus was not assessed in this study.) This finding, which corroborates the previous finding that the majority of HSV-2 infections remain undiagnosed [1, 2], highlights the importance of improving awareness and recognition of genital herpes among pregnant women. That all women who tested positive for HSV-2 agreed that it is important to use medicine to treat herpes during pregnancy and were willing to take a medicine during the last 4 weeks of pregnancy to treat herpes suggests that women aware of their seropositive status will implement measures to reduce the risk of transferring the virus to their infants.

Importantly, 31% of population was seronegative for both HSV-1 and HSV-2 and therefore susceptible to getting primary genital herpes during pregnancy. The majority of neonatal HSV infections occur among infants born to women experiencing primary HSV infections. The risk of neonatal infection is particularly high when the mother contracts a primary or nonprimary first episode of genital herpes late in gestation [23]. These considerations underscore the importance of education of women and their partners about the prevention of acquisition of HSV during pregnancy.

## 5. Conclusions

Results of this study corroborate previous findings on the high prevalence of HSV seropositivity among pregnant women and support the feasibility of HSV serologic testing and counseling in this population. Further research is needed on methods of counseling patients about HSV and neonatal herpes and on specific information that should be included in counseling materials.

## Figures and Tables

**Table 1:** Demographics and sexual history.

	Sample (*N* = 304)	Subgroup willing to be tested (*n* = 134)	Subgroup unwilling to be tested (*n* = 169)

Age, mean (SD), years	29.3 (6.2)	29.3 (6.1)	29.3 (6.3)
Race, %			
White	87	84	91
Black	8	7	8
Asian	5	7	2

Type of insurance,* %			
Medicare	0	0	0
Medicaid	16	10	21
Private/employer-provided	83	89	79
None	<1	<1	0

Marital status,* %			
Single, living alone	5	2	8
Single, living with partner	25	23	27
Single, living with roommates/parents	10	11	9
Married	59	65	54
Divorced	1	0	2
Widowed	0	0	0

Employment status, %			
Full time	40	39	41
Part Time	24	21	27
Unemployed	14	14	14
Homemaker/housewife	16	16	15
Full-time student	5	7	2
Other	3	4	3

Highest level of education, %			
Elementary	<1	0	1
High school	31	28	34
Some college	32	29	34
Technical/other professional school	5	5	4
College	20	22	18
Postgraduate	12	16	9

2004 household pretax income, %			
<$20,000	17	14	20
$20,000 to $39,999	26	29	23
$40,000 to $59,999	23	18	26
$60,000 to $79,999	13	15	12
$80,000 to $99,999	10	12	9
≥$100,000	10	12	9

Age at first sexual intercourse, %			
15 or younger	20	19	20
16 or older	80	81	80

Estimated # of sexual partners, %			
1	17	19	15
2 to 4	43	40	47
5 to 9	24	25	24
10 to 19	13	13	13
20 to 29	1	2	<1
30 to 39	<1	<1	1

*P < .05 for subgroup willing to be tested versus subgroup not willing to be tested.

**Table 2:** Summary of precounseling reasons for choosing not to be tested for HSV-2 on Visit 1. Data are expressed as (%) of participants.

	Subgroup changing from willing to unwilling (*n* = 115)	Subgroup unwilling to be tested (*n* = 169)	Subgroup never willing (*n* = 52)
Cost of the test	*n* = 72	*n* = 126	*n* = 52
Strongly disagree/disagree	100	99	67
Agree/strongly agree	0	1	33

Fear of results	*n* = 72	*n* = 126	*n* = 52
Strongly disagree/disagree	77	73	69
Agree/strongly agree	23	27	31

Knowing is not important to me	*n* = 72	*n* = 126	*n* = 52
Strongly disagree/disagree	99	89	77
Agree/strongly agree	1	11	23

Concern about what will be done with results	*n* = 72	*n* = 126	*n* = 52
Strongly disagree/disagree	40	49	59
Agree/strongly agree	60	51	41

No knowledge of genital herpes	*n* = 72	*n* = 126	*n* = 52
Strongly disagree/disagree	100	95	95
Agree/strongly agree	0	5	10

No knowledge of how genital herpes will affect my baby	*n* = 72	*n* = 126	*n* = 52
Strongly disagree/disagree	100	98	96
Agree/strongly agree	0	2	4

Not at risk for genital herpes	*n* = 71	*n* = 125	*n* = 52
Strongly disagree/disagree	9	15	22
Agree/strongly agree	91	85	78

No benefit to finding out results	*n* = 72	*n* = 126	*n* = 52
Strongly disagree/disagree	75	78	82
Agree/strongly agree	25	22	18

Test would take too much time	*n* = 72	*n* = 126	*n* = 52
Strongly disagree/disagree	4	66	92
Agree/strongly agree	53	34	8

Being tested is too personal	*n* = 72	*n* = 125	*n* = 51
Strongly disagree/disagree	34	47	65
Agree/strongly agree	66	53	35

Having to tell my partner the test results	*n* = 72	*n* = 126	*n* = 52
Strongly disagree/disagree	83	66	65
Agree/strongly agree	17	24	35

**Table 3:** HSV seroprevalence. Data are expressed as number (%) of participants.

	*N* = 134
HSV-1 positive	81 (60)
HSV-2 positive	15 (19)

HSV-1/HSV-2 combinations	

HSV-1 positive and HSV-2 positive	15 (11)
HSV-1 positive and HSV-2 negative	66 (49)
HSV-1 negative and HSV-2 positive	12 (9)
HSV-1 negative and HSV-2 negative	41 (31)

**Table 4:** Summary of the Understanding Counseling Questionnaire. Data are expressed as (%) of participants answering the true/false questions correctly.

	Sample (*N* = 303)	Subgroup willing to be tested (*n* = 134)	Subgroup unwilling to be tested (*n* = 169)
Visit 1			

Genital herpes is a contagious virus spread by intimate skin-to-skin contact.	95	94	96
Genital herpes is a very common disease.	97	>99	96
You cannot spread herpes to your newborn at the time of delivery.	85	85	86
Even if you have genital herpes, it is still possible to have a healthy baby.	97	97	97
There is no test you can take to find out if you have the virus.	95	94	95
There is no cure for genital herpes.	(*n* = 302) 86	87	(*n* = 168) 85
HSV-1 (the cold sore virus) can cause genital herpes.	70	68	71

Visit 2	HSV seronegative participants (*n* = 107)		

Genital herpes cannot be spread by intimate skin-to-skin contact.	86		
Genital herpes can be active without any visible signs or symptoms of an outbreak.	98		
If you get genital herpes late in your pregnancy, your baby will have a high risk of getting herpes.	86		
Even though you have tested negative, it is still possible that your partner has genital herpes.	100		
You can reduce your risk of getting herpes by practicing safer sex.	100		
If your partner has genital herpes, he can take medicine to treat his herpes and reduce the risk of spreading it to you.	95		
If you get HSV-1 (the cold sore virus) genital infection during your pregnancy, you can pass it on to your baby.	75		

Visit 2	HSV seropositive participants (*n* = 27)		

Herpes is less common than diabetes or asthma.	92		
Once diagnosed, it is difficult to know how long you have had genital herpes.	96		
Genital herpes can be treated to reduce the risk of having outbreaks.	100		
You cannot spread genital herpes to your newborn at the time of delivery.	85		
There are things you can do to reduce the risk of spreading genital herpes to your baby at the time of delivery.	100		
Genital herpes can be cured.	88		
If you get HSV-1 (the cold sore virus) genital infection during your pregnancy, you can pass it on to your baby.	81		

## Appendix

### Counseling Script for Visit 1

What Is Genital Herpes?

- Genital herpes is a virus that is spread through intimate, skin-to-skin contact, including vaginal, oral, or anal sex.
- The genital herpes virus can be active on the surface of the skin with symptoms and a visible outbreak or without any visible signs or symptoms.
- There are many symptoms that can be associated with the virus such as itching, redness, bumps, tingling, and cuts in the genital area/boxer short area.
- It is possible that what you think are yeast infections, urinary tract infections, or vaginal discharge actually may be cases of genital herpes.
- Remember, there is no cure for herpes. However treatment options are available to treat this disease.

Who Has Genital Herpes?

- One in five Americans over the age of 14 has the genital herpes virus, that is approximately 45 million people.
- And when you consider that among American adults who have the genital herpes virus, 9 out of 10 (40 million) do not even know they have it.
- Anyone who is sexually active—even with just one person—can get GH; there are up to one million new cases each year.
- You may want to find out if your symptoms are actually what you think they are.

Why Is It Important to the Pregnant Woman?

- If you have the genital herpes virus, you are at a risk of spreading herpes to your newborn at the time of delivery.

What Are the Risks to the Baby?

- If the baby gets the herpes virus, also called neonatal herpes, the baby usually gets sick during the first one to two weeks of life.
- This illness can cause mental and physical disabilities and in the worst cases death.
- But remember even if you have genital herpes, you can still have a healthy a baby.
- There are things that you can do to reduce the risks to your baby, but first you need to know if you have the genital herpes virus.

What Is the Purpose of a Blood Test for Genital Herpes?

- As mentioned, the first step is to take the test so that you are aware of whether or not you have herpes.
- If you take the test today, at your next visit we will be able to tell you the results of the test.
- Even if you think you do not have the virus, it is important to take the test to determine that you *in fact do not have herpes*, then you can take steps to reduce the risk of getting herpes, *especially during your pregnancy*.

What Does It Mean If My Test Is Negative?

- If your test shows that you are negative, you do not have herpes but if you were only recently exposed (within the last 3 months) a repeat test may be needed.
- It also means that it is important for you to take steps to keep from getting the virus during your pregnancy:
  - know if your partner is infected,
  - practice safer sex including use of condoms.

What Does It Mean If My Test Is Positive?

- If your test shows that you are positive, you do have herpes.
- As with any medical test, there is a small chance the positive result is a false positive. If you need to confirm the results, there are different tests that can be done.
- Remember, genital herpes is more common than diseases like diabetes and asthma. So if you have herpes, you are not alone, and you should not feel embarrassed or ashamed.
- If you do have this virus, there are things you can do for yourself and your baby.

What Is the Importance of the Cold Sore Virus (HSV-1)?

- HSV-1 (the cold sore virus) can also cause a genital herpes infection by being spread during oral-genital sex.
- Women who have HSV-1 have little to no risk for spreading HSV-1 to their baby because they have developed protective antibodies to this virus that they pass to their baby.
- If you do not have HSV-1, it is important for you to take steps to keep from getting HSV-1 during pregnancy:
  - know if your partner has HSV-1,
  - abstain from oral-genital sex during your pregnancy.

## References

[1] Xu F, Sternberg MR, Kottiri BJ (2006). Trends in herpes simplex virus type 1 and type 2 seroprevalence in the United States. *Journal of the American Medical Association*.

[2] Fleming DT, Mcquillan GM, Johnson RE (1997). Herpes simplex virus type 2 in the United States, 1976 to 1994. *The New England Journal of Medicine*.

[3] Xu F, Markowitz LE, Gottlieb SL, Berman SM (2007). Seroprevalence of herpes simplex virus types 1 and 2 in pregnant women in the United States. *American Journal of Obstetrics and Gynecology*.

[4] Baker DA (2005). Antiviral therapy for genital herpes infections in pregnancy. *Expert Review of Anti-Infective Therapy*.

[5] Kimberlin DW (2005). Herpes simplex virus infections in neonates and early childhood. *Seminars in Pediatric Infectious Diseases*.

[6] Whitley R, Davis EA, Suppapanya N (2007). Incidence of neonatal herpes simplex virus infections in a managed-care population. *Sexually Transmitted Diseases*.

[7] Kimberlin DW (2004). Neonatal herpes simplex infection. *Clinical Microbiology Reviews*.

[8] (2007). Management of herpes in pregnancy. ACOG practice bulletin no. 82. *Obstetrics & Gynecology*.

[9] Gardella C, Barnes J, Magaret AS, Richards J, Drolette L, Wald A (2007). Prenatal herpes simplex virus serologic screening beliefs and practices among obstetricians. *Obstetrics and Gynecology*.

[10] Brown ZA, Gardella C, Wald A, Morrow RA, Corey L (2005). Genital herpes complicating pregnancy. *Obstetrics and Gynecology*.

[11] Brown Z (2004). Preventing herpes simplex virus transmission to the neonate. *Herpes*.

[12] Brown ZA (2000). HSV-2 specific serology should be offered routinely to antenatal patients. *Reviews in Medical Virology*.

[13] Baker D, Brown Z, Hollier LM (2004). Cost-effectiveness of herpes simplex virus type 2 serologic testing and antiviral therapy in pregnancy. *American Journal of Obstetrics and Gynecology*.

[14] Baker DA (2005). Risk factors for herpes simplex virus transmission to pregnant women: a couples study. *American Journal of Obstetrics and Gynecology*.

[15] Kinghorn GR (2002). Debate: the argument for—should all pregnant women be offered type-specific serological screening for HSV infection?. *Herpes*.

[16] Arvin AM (2002). Debate: the argument against—should all pregnant women be offered type-specific serological screening for HSV infection?. *Herpes*.

[17] Tita ATN, Grobman WA, Rouse DJ (2006). Antenatal herpes serologic screening: an appraisal of the evidence. *Obstetrics and Gynecology*.

[18] Thung SF, Grobman WA (2005). The cost-effectiveness of routine antenatal screening for maternal herpes simplex virus-1 and -2 antibodies. *American Journal of Obstetrics and Gynecology*.

[19] Rouse DJ, Stringer JSA (2000). An appraisal of screening for maternal type-specific herpes simplex virus antibodies to prevent neonatal herpes. *American Journal of Obstetrics and Gynecology*.

[20] Barnabas RV, Carabin H, Garnett GP (2002). The potential role of suppressive therapy for sex partners in the prevention of neonatal herpes: a health economic analysis. *Sexually Transmitted Infections*.

[21] Vonau B, Low-Beer N, Barton SE, Smith JR (1997). Antenatal serum screening for genital herpes: a study of knowledge and attitudes of women at a central London hospital. *British Journal of Obstetrics and Gynaecology*.

[22] Edmiston N, O’Sullivan M, Charters D, Chuah J, Pallis L (2003). Study of knowledge of genital herpes infection and attitudes to testing for genital herpes among clinic attendees. *Australian and New Zealand Journal of Obstetrics and Gynaecology*.

[23] Brown ZA, Benedetti J, Selke S, Ashley R, Watts DH, Corey L (1996). Asymptomatic maternal shedding of herpes simplex virus at the onset of labor: relationship to preterm labor. *Obstetrics and Gynecology*.

